# PDAUG: a Galaxy based toolset for peptide library analysis, visualization, and machine learning modeling

**DOI:** 10.1186/s12859-022-04727-6

**Published:** 2022-05-28

**Authors:** Jayadev Joshi, Daniel Blankenberg

**Affiliations:** 1 Genomic Medicine Institute, Lerner Research Institute, Cleveland Clinic, Cleveland, OH USA; 2 Department of Molecular Medicine, Cleveland Clinic Lerner College of Medicine, Case Western Reserve University, Cleveland, OH USA

## Abstract

**Background:**

Computational methods based on initial screening and prediction of peptides for desired functions have proven to be effective alternatives to lengthy and expensive biochemical experimental methods traditionally utilized in peptide research, thus saving time and effort. However, for many researchers, the lack of expertise in utilizing programming libraries, access to computational resources, and flexible pipelines are big hurdles to adopting these advanced methods.

**Results:**

To address the above mentioned barriers, we have implemented the peptide design and analysis under Galaxy (PDAUG) package, a Galaxy-based Python powered collection of tools, workflows, and datasets for rapid in-silico peptide library analysis. In contrast to existing methods like standard programming libraries or rigid single-function web-based tools, PDAUG offers an integrated GUI-based toolset, providing flexibility to build and distribute reproducible pipelines and workflows without programming expertise. Finally, we demonstrate the usability of PDAUG in predicting anticancer properties of peptides using four different feature sets and assess the suitability of various ML algorithms.

**Conclusion:**

PDAUG offers tools for peptide library generation, data visualization, built-in and public database peptide sequence retrieval, peptide feature calculation, and machine learning (ML) modeling. Additionally, this toolset facilitates researchers to combine PDAUG with hundreds of compatible existing Galaxy tools for limitless analytic strategies.

**Supplementary Information:**

The online version contains supplementary material available at 10.1186/s12859-022-04727-6.

## Introduction

Interest in peptides-related research has been gaining in popularity over the last several decades [35]. A large number of naturally occurring peptides (over 7000) with potentially important roles in human physiology have been identified. Currently, more than 140 peptide therapeutics are in different stages of clinical trials [17]. In view of their integral importance in a number of signal transduction pathways, they are ideal candidates for functioning as drugs, especially as anticancer or antimicrobial agents [1]. Usually, peptides are naturally occurring molecules that are synthesized by cellular processes and adopt alternative conformations according to their biological functions [35]. Peptides can either act as natural ligands in the form of cofactors, coenzymes, and hormones, or directly interact with macromolecules including proteins, RNA, or DNA [15]. The research underlying the design of therapeutic peptides, such as peptide-based drugs and vaccines, demands intense effort and assets for establishing their pharmacokinetic and pharmacodynamic properties such as serum stability, bioavailability toxicity, etc. [7, 44]. Peptide-based vaccines have emerged as a powerful approach to counter infectious diseases and cancer [37]. Characterization of peptides that bind to specific major histocompatibility complex (MHC) molecules is therefore of great importance for peptide-based vaccines. However, in comparison to expensive and lengthy biochemical experiments, bioinformatics methods for predicting MHC binding peptides have been very popular in recent years [24, 28, 45]. Various computational approaches have been shown to offer the best cost–benefit ratio across translational research areas [50, 59, 60]. Leveraging in-silico approaches to uncover peptides with desired pharmacological action can be expected to significantly lower the cost and time required to establish a drug or a vaccine candidate [34]. In fact*,* computational predictions of peptides with desired functions have been providing effective alternatives to traditional methods in peptide research, thus saving time and effort [5, 22, 25, 33, 39, 52]. The concept of prioritizing sequence-based properties of a protein sequence as a function of sequence-derived features is not new [29]. Over the past decade, approaches based on physicochemical, compositional properties, k-mer counting, etc. have been proposed [10, 51, 62]. With the rise of computational power, feature-based methods have evolved substantially, expanding into the analysis of 3D structure level of biomolecules [23]. However, necessary programming and mathematics expertise, as well as limitations in hardware resources, are among the core challenges associated with utilizing programming-based resources [30, 49]. Web-based data analysis platforms, such as Galaxy [2, 19, 26], have been providing a user-friendly solution to enable researchers to include advanced data analysis methods in their work. Galaxy is an open-source, web-based platform for accessible, reproducible, and transparent computational research. It provides a wealth of computational tools, workflows, and training materials for advance data visualization and analysis.

In this paper, we present PDAUG, a Galaxy tool suite that includes 24 different tools for the analysis of peptide libraries. The main objective of this paper is to provide a set of user-friendly tools for peptide library generation, visualization, machine learning (ML) modeling and analysis. PDAUG provides user-friendly tools in various categories including peptide library generation, feature analysis, data visualization and plotting, ML modeling, and dataset retrieval. These modular command-line tools leverage the Galaxy platform to provide an interactive graphical interface for each tool as well as an expandable set of workflows for peptide data representation and analysis. Individual tools rely on pandas dataframes to handle the data matrices, with tabular and FASTA formats for input/output (IO) operations. Data formats were chosen for PDAUG to complement the strengths of Galaxy’s existing toolsets and to enhance usability.

Tests have been defined for each tool to maintain reliable and reproducible results. In addition, we have produced an interactive Galaxy tutorial for each example workflow used in this article, which demonstrates the functionality and usability of this toolset. Finally, we utilized this toolset to assess a suitable combination of features and ML algorithms in predicting the anti-cancer properties of peptides to demonstrate the usability of this toolset in peptide research.

## Implementation

**Fig. 1:**
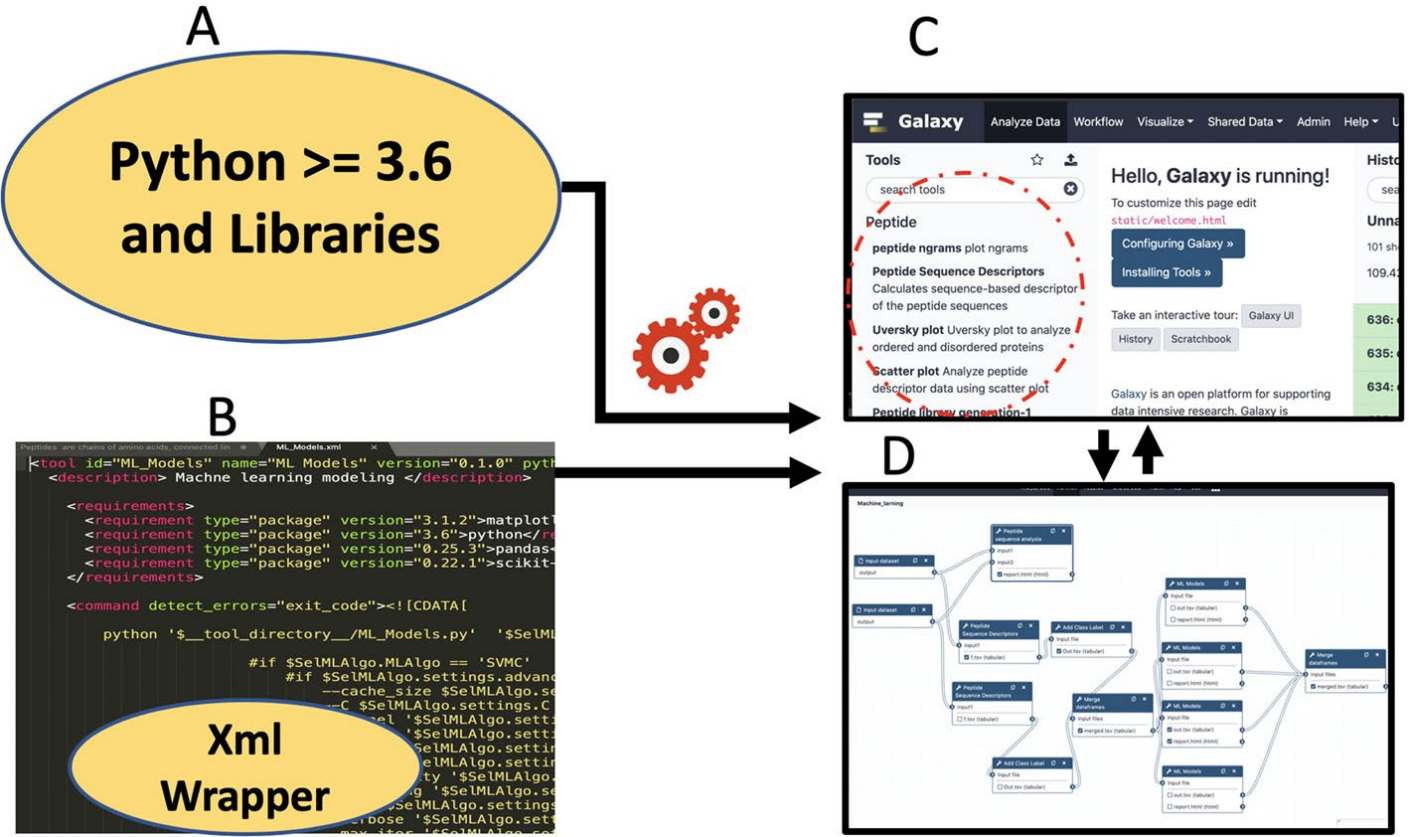
Extending peptide library analysis with the PDAUG toolset inside Galaxy. **A** Tools are created with Python libraries. **B** Implementing Galaxy tool wrappers and tests for each tool. **C** PDAUG toolset with 24 individual tools. **D** Implementing reusable workflows using PDAUG

**Fig. 2:**
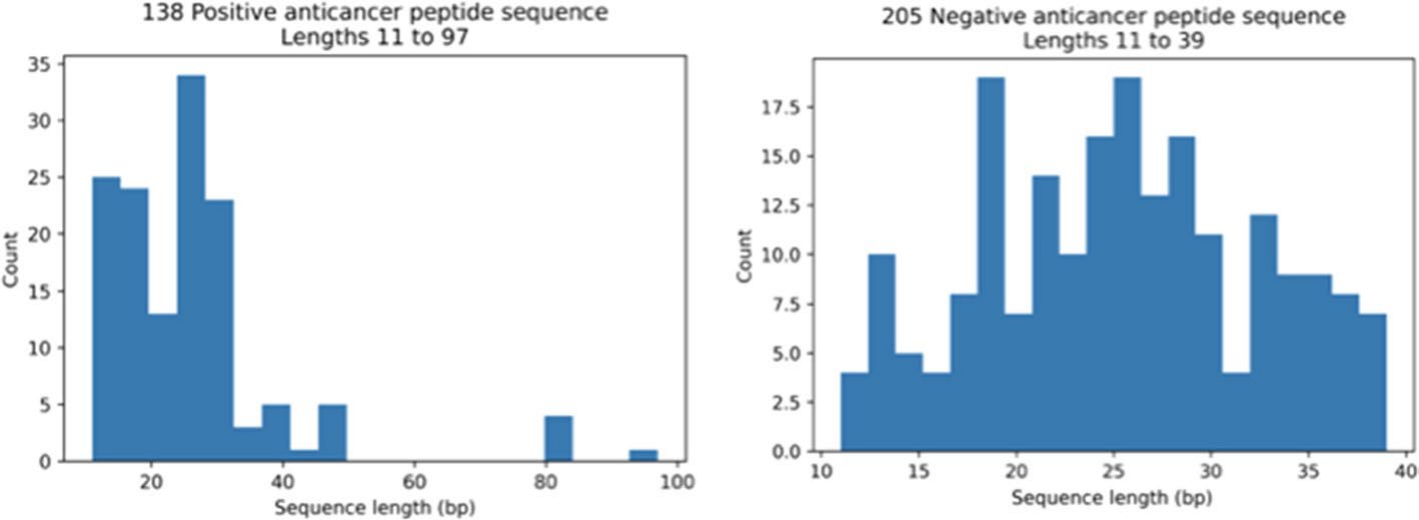
Sequence length distributions for the anticancer peptide and non-anticancer peptides. Mean lengths of anticancer and non-anticancer peptides are 40.06 and 32.25 AA, respectively, with less variability in length shown among the anticancer peptides

A graphical overview of PDAUG has been described in Fig. 1. Galaxy tools depend on two components, (1) an underlying software dependency, usually programming scripts or command-line tools that perform all the algorithmic tasks under the hood, Fig. 1A, and (2) an extensible markup language (XML) wrapper that describes the user interface and contains the commands to execute software tools. Figure 1B. By default, Galaxy automatically generates a separate conda environment for each combination of underlying dependencies for every tool, ensuring versioned reproducibility. All other complex tasks, such as job submission, database management, web-server, workflow, etc., are handled by the Galaxy platform, but can be delegated to third-party resources by an administrator, Fig. 1C, D. PDAUG tools are categorized into 9 different categories based on their functionalities. Implementation details for each tool have been included in Table 1, and important Python packages have been highlighted.

**Table 1:** Description of PDAUG tools. PDAUG toolset comprises 24 different tools across 9 functional categories

Functionality	Tool name	Major libraries used
Data visualization and plotting	PDAUG basic plots	matplotlib*, pandas*, seaborn*, quantiprot
	PDAUG fishers plot	
	PDAUG peptide data plotting	
	PDAUG peptide Ngrams	
	PDAUG sequence network	
	PDAUG peptide length distribution	
	PDAUG uversky plot	
Descriptor calculation	PDAUG AA property based peptide descriptor	modlAMP, pandas, pydpi
	PDAUG peptide core descriptors	
	PDAUG peptide global descriptors	
	PDAUG sequence property based descriptors	
	PDAUG word vector descriptor	
Peptide library generation	PDAUG AA property based peptide generation	modlAMP, pandas
	PDAUG sequence based peptide generation	
ML	PDAUG ML models	sklearn*, matplotlib, seaborn, pandas, gensim, nltk
	PDAUG word vector model	
Circular dichroism (CD) data analysis	PDAUG peptide CD spectral analysis	modlAMP, pandas
Peptide 3D structure	PDAUG peptide structure builder	fragbuilder, pandas
Core functionality	PDAUG peptide sequence analysis	modlAMP, pandas
	PDAUG peptide core functions	
Peptide data access	PDAUG peptide data access	modlAMP, biopython, pandas
Data handling and IO	PDAUG TSVtoFASTA	pandas
	PDAUG merge dataframes	
	PDAUG AddClassLabel	

Libraries utilized for functionally important tasks are listed for each tool

*Python libraries used in data science

### Programming languages

Due to its popularity among the scientific community, Python has been chosen to implement the functions and backend scripts for these Galaxy tools. We have leveraged popular scientific libraries such as NumPy, SciPy, pandas, Matplotlib, scikit-learn (sklearn), etc. for data manipulation and representation to maintain uniformity and simplicity. Galaxy tool wrappers have been designed and uploaded to the ToolShed [6], enabling point-and-click installation. We also provide a Docker image containing Galaxy and these tools pre-installed (https://hub.docker.com/r/jayadevjoshi12/galaxy_pdaug, https://github.com/jaidevjoshi83/docker_pdaug).

### Accessing peptide data from pre-populated local and remote web-based resources

In addition to allowing the upload of user-provided datasets, PDAUG has been equipped with the “PDAUG Peptide Data Access” tool for quick and easy access to various publicly available peptide datasets. This tool is implemented based on modlAMP [40] and Biopython [12], and includes antimicrobial peptides (AMPs), trans-membrane peptides, peptides from the UniProt database, anticancer peptides (ACPs), helical transmembrane peptides (HTPs) and randomly scrambled AMPs. Additionally, options have been provided to fetch data directly from two popular web resources: the antimicrobial peptide database (APD) [57] and the collection of antimicrobial peptides database (CAMP) [56].

### Peptide library generation

Two tools with several options to generate peptide sequences have been implemented. These tools provide various methods based on amino acid (AA) and sequence properties to generate peptide sequence libraries. These peptides with different properties can be utilized for further analysis inside the Galaxy.

1. *PDAUG AA Property-Based Peptide Generation*. This tool generates sequences mostly based on AA properties. The user can generate peptide sequences based on 10 different options including, "AmphipathicArc Peptides'' which returns peptides with presumed amphipathic helices, "AMPngrams Peptides " which returns peptides from the most frequent n-grams in the APD3 database, "Centrosymmetric Peptides'' which returns centrosymmetric peptide sequences with a symmetry axis, "Helices Peptides" which returns presumed amphipathic helical peptides, "HelicesACP Peptides" which returns peptides with AA probability similar to helical ACPs, "Kinked Peptides'' which returns peptides with presumed amphipathic helices with a kink, "Hepahelices Peptides" which returns peptides with presumed amphipathic helices and a heparin-binding-domain, "Oblique Peptides" which returns presumed oblique oriented peptides, "Random Peptide", which returns random peptides with a specified AA distribution, and "MixedLibrary Peptides " which returns a library of mixed peptides. Most of the functions of this tool have been implemented on top of modlAMP [40] and pandas libraries.
2. *PDAUG Sequence-Based Peptide Generation*. This tool generates peptide libraries based on three different options. The primary method “Random Peptides” is based on permutation and combinations that perform a search for all the possible combinations of 20 AA within the given length. The second method “Mutated Peptides” produces the replacement of existing AA with the remaining 19 possibilities at given positions. The last method “Sliding Window Peptides” takes an input of a protein sequence and generates random peptide fragments based on a sliding window and fragment size.

### Peptide structure

The tool “PDAUG Peptide Structure Builder” has been implemented to generate a peptide structure based on the libraries FragBuilder [11] and Open Babel [42]. This tool can generate peptide sequences of up to 4 AA, which can then further be utilized in small peptide docking simulations with molecular docking tools inside of Galaxy such as AutoDock Vina [54].

### Peptide descriptor generation

Four different tools have been implemented that calculate more than 10,000 descriptors based on 50 different classes of peptide descriptors for a given peptide sequence. We have included mathematical details in the Additional file 11: Table S1.

1. PDAUG Peptide Global Descriptors. This tool calculates simple one-dimensional peptide descriptors based on 11 different options which include Sequence Length, Molecular Weight, Sequence Charge, Charge Density, Isoelectric Point, Instability Index, Aromaticity, Aliphatic Index, Boman Index, Hydrophobic Ratio and All. These descriptors are important to define the global properties of a peptide sequence and can be utilized to build ML models to predict biological properties.
2. *PDAUG Sequence Property-Based Descriptors*. This tool calculates descriptors based on 13 different options. Option "GetAAComp" calculates AA composition descriptors, "GetDPComp" calculates dipeptide composition descriptors "GetTPComp" calculates tri-peptide composition descriptors, "GetMoreauBrotoAuto" calculates normalized Moreau-Broto autocorrelation descriptors, "GetMoranAuto calculates" moran autocorrelation descriptors, "GetGearyAuto" calculates Geary autocorrelation descriptors, "GetCTD" calculates composition Transition Distribution descriptors, "GetPAAC" calculates Type I Pseudo AA composition descriptors, "GetAPAAC" calculates amphiphilic (Type II) Pseudo AA composition descriptors, "GetSOCN" calculates sequence order coupling numbers, "GetQSO" calculates quasi sequence order descriptors, "GetTriad" calculates the conjoint triad features from the protein sequence and, “BinaryDescriptor” calculates the binary descriptor of peptides with identical lengths. Lastly, the “All” option calculates all the above descriptors, excluding binary, with one click. These descriptors are implemented based on the PyDPI library [8].
3. *PDAUG AA Property-Based Peptide Descriptor*. This tool calculates descriptors derived from AA properties based on six different options. "Calculate AutoCor" computes descriptors via auto-correlating the AA values. "Calculate CrosCor" computes descriptors via cross-correlating the AA values. "Calculate Movement" computes a descriptor based on the maximum or mean movement of the AA values. The "Calculate Global” option computes descriptors via calculating global/window averaging descriptor values. “Calculate Profile” computes descriptors via calculating hydrophobicity or hydrophobic moment profiles for given sequences and fitting for slope and intercept. “Calculate Arc” computes descriptors via calculating property arcs. These descriptors depend upon the given descriptor scale and window size.
4. *PDAUG Word Vector Descriptor*. Word2vec is a popular technique of word embedding [38, 3] and shows a better performance in protein and peptide classification over other sequence descriptors [22, 58, 61]. In this toolset, we have included two tools. The first tool, "PDAUG Word Vector Model", generates a word2vec model that contains the contextual information for each trigram in the corpus of given protein sequences. Input protein sequences are referred to as corpus and are utilized to generate a trigram-based vocabulary. Gensim library [48] is used to apply a continuous bag of words (CBOW) or skip-gram algorithm to generate a 200-dimensional vector for each trigram. These 200-dimensional vectors represent the context information of all the trigrams present in the training. These vectors can be utilized to generate the descriptor for peptides using the second tool, “PDAUG Word Vector Descriptor”. A pre-calculated skip-gram word2vec model, generated based on the UniProtKB/TrEMBL database [22], has been provided with the supplementary data as model.txt, which can be utilized directly with the “PDAUG Word Vector Descriptor” tool to calculate 200 descriptors.

### Data visualization and analysis

PDAUG contains several data visualization tools for both sequence and feature-based data representations.

1. *PDAUG Basic Plots*. This tool is equipped with four different options to plot the data in tabular and FASTA formats. Four different options, "Heat Map", "Box Plot", "Scatter Plot", and "Word Cloud" have been provided for standard data visualization.
2. *PDAUG Fisher’s Plot*. Fisher’s plot has been implemented to assess two peptide sequences based on their feature spaces. In principle, Fisher's plot compares two peptide sequences in two-dimensional spaces, defined by quantitative features of peptide sequences. This tool computes Fisher’s exact test on a local and global ratio of peptide sequence in a feature space where the global and local ratio is computed either in the whole feature space or in a feature space belonging to each set. This tool is implemented based on the Quantiprot [32] package and can be utilized to compare two peptide libraries.
3. *PDAUG Peptide Data Plotting*. Four different plotting options have been provided in this tool. The “Helical Wheel” option plots a helical wheel plot for a given peptide sequence. The “Probability Density Estimation” option plots probability density estimations of given data vectors. The “Violin Plot” option creates a violin plot from the given data array. The “Amino Acid Distribution” option plots the amino acid distribution of a given sequence library.
4. *PDAUG Peptide Ngrams*. Distribution of n-grams varies from sequence to sequence with different AA compositions that affect the property of peptide sequences. This tool counts n-grams in the entire peptide sequence data and fits their distribution with Zipf’s law, also known as the power-law distribution [32].
5. *PDAUG Sequence Similarity Network*. This tool calculates the Levenshtein distance between peptide sequences, and plots the data in the form of a sequence similarity network. A dispersed and multiply-clustered network represents less similarity between sequences. Conversely, a network that is compact and has a smaller number of clusters represents high sequence similarity between sequences [4].
6. *PDAUG Uversky Plot*. The Uversky plot separates proteins into globular and intrinsically disordered protein subsets on the basis of their mean net charge versus mean hydropathy [55]. Uversky plot has been implemented under the tool name “PDAUG Uversky Plot”, where users can compare two different peptide libraries on the basis of their globular and intrinsically disordered properties [32].
7. *Summary Plot*. Summary plot options of the “PDAUG Peptide Sequence Analysis” tool consist of six subplots for an overall summary of peptide libraries based on AA fractions, global charge fraction, sequence length distribution, global hydrophobicity, and hydrophobic movement.
8. *PDAUG Peptide Core Functions*. This tool is equipped with four options. The “Mutate Amino Acids” option randomly mutates amino acids at the several positions per sequence with a given probability value. The “Filter Duplicates” option removes duplicate sequences from a library. The “Keep NaturalAA” option filters out sequences with unnatural AA. And the “Filter Amino Acids” option filters out sequences with user-specified AA.
9. *PDAUG Peptide Sequence Analysis*. This tool provides functionality to calculate several important sequence-based properties such as AA frequency, global hydrophobicity, hydrophobic moments, total molecular charge, and sequence length.

### ML model building, cross-validation, and accuracy assessment

PDAUG provides standard utilities for ML modeling and model selection via “PDAUG ML Models” tools. These tools can classify peptides in a binary fashion and predict different peptide classes. A total of seven different supervised algorithms and one artificial neural network algorithm have been implemented. These include logistic regression classifier (LRC) [53], Gaussian Naïve–Bayes classifier (GNBC) [18], K-nearest neighbor classifier (KNBC) [14], decision-tree classifier (DTC) [27], Support vector machines classifier (SVMC) [13], random forest classifier (RFC) [36], gradient boosting classifier (GBC) [41], Stochastic gradient descent classifier (SGDC) [63], and multilayer perceptron (MLP) [46]. Cross-validation has been included in our methodology for accuracy estimation [31]. The Performance of an ML algorithm is commonly assessed by several metrics. (a) *Precision*, also known as the probability of positive values (PPV), is summarized as the probability of currently predicted positive instances and estimated on the basis of true positive (TP) and False positive (FP). (b) *Recall*, also known as sensitivity, is defined as the estimation of the percentage of the correctly predicted positive instances and is also calculated with TP and FP. (c) *F1-measure* is also an important estimate of model accuracy and can be defined as a harmonic mean of precision. The value for each of these three estimates falls between 0 and 1, with larger values indicating better performance and better accuracy. (d) *Accuracy* is described as correctly predicted instances and calculated on the basis of TP and true negative (TN) divided by TP, TN, FP, and false negative (FN). (e) AUC is Area under the ROC curve, where ROC is a receiver operating characteristic. AUC represents the area covered by ROC.

Two normalization methods, max–min and z-scaling, to optionally normalize the data before computational modeling is also implemented. Four different options, “Internal Test”, “Train-Test Split”, “External Test Data”, which enables the inclusion of a separate data file as a testing data set other than training data, and “Predict unknown” have been implemented to test generated models.

### Circular dichroism spectral data analysis

Circular dichroism (CD) is a technique used to potentially determine the secondary structure and folding properties of proteins [47]. We have included “PDAUG Peptide CD Spectral Analysis”, a tool based on the modlAMP and pandas libraries, that can be used to analyze CD spectroscopy of peptides data in different solvents. This tool handles CD data based on 4 different options. The “Calculate Ellipticity” option calculates molar ellipticity and mean residue ellipticity for all the tabular data provided. The “Generate CD Plots” option generates the CD plots. The “Save Data in DichroWeb Readable Format" option converts and returns data into DichroWeb compatible format. The “Calculate the Percent of Helicity” option calculates percentage of helicity based on mean residue ellipticity data.

### IO operations

These tools were implemented based on the pandas dataframe for seamless data operation between the various tools. We have included three tools to handle and manipulate FASTA and tabular data files. “PDAUG TSVtoFASTA” tool changes the input data formats from tabular to FASTA and splits the data on the basis of their class label in separate FASTA files. The “PDAUG AddClassLabel” tool adds the desired class label to the samples in a dataframe. The last tool in this category is “PDAUG Merge Dataframes” which merges two user-provided dataframes. This simplifies IO operations allowing PDAUG to interact with other existing Galaxy tools.

### ML workflow for the example dataset

A high-quality peptide dataset was extracted from a previously published work [21]. Four different types of descriptor sets have been used to construct ML models which include (1) Composition, Transition, Distribution descriptors (CTD), (2) Geary autocorrelation descriptors (GearyAuto), (3) Moran autocorrelation descriptors (MoranAuto), and (4) Word Vector descriptor. A total number of 140, 200, 200, and 200 descriptors have been calculated, respectively.

Six ML algorithms, LRC, RFC, GBC, DTC, SGDC, and SVMC, have been applied to the training dataset and tenfold cross-validation was used for accuracy estimation (Kohavi 1995). Data normalization was applied to the data before ML modeling and the effect of normalization was assessed on ML models. The entire workflow was applied to the four descriptor sets and the performance was estimated based on accuracy, precision, recall, f1-score, and AUC scores.

## Result and discussion

PDAUG has been developed for peptide library analysis to meet the increasing popularity of the design and screening of large peptide libraries. Traditional peptide library design and analysis is labor-intensive work that requires bioinformatics methods to enable scalable alternatives. To maximize accessibility and impact, we have released PDAUG as a set of Galaxy tools, enabling web-based access and sharing of tools, pipelines, and analysis results. For more details such as functionality and implementation details please refer to Table 1.

### ML modeling of example anticancer/non-anticancer peptide dataset

To demonstrate the usability of the PDAUG toolset, we have presented a case study in which ML models have been built that can predict peptides with anti-cancer properties. We have collected a high-quality dataset that was extracted from a previously published work [21]. Initial data contains 192 anticancer peptides ACPs and 215 non-ACPs. The example dataset was cleaned as described in previously published work [52] in order to remove redundancy and improve the data quality. Finally, a total number of 138 ACPs and 138 non-ACP sequences were selected for the final training data set.

**Fig. 3:**
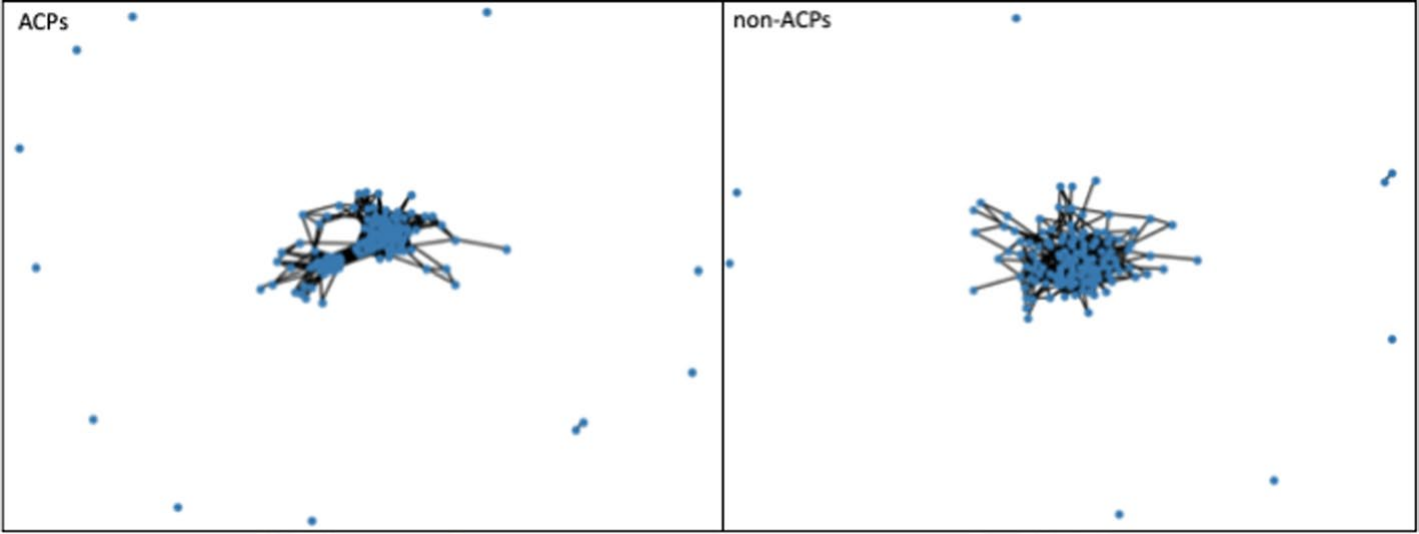
Sequence similarity network of the ACPs and non-ACPs. In comparison to the non-ACPs peptides, ACPs show two compact clusters that indicate a relatively high sequence similarity. In case of non-ACPs, relatively scattered networks have been observed

**Fig. 4:**
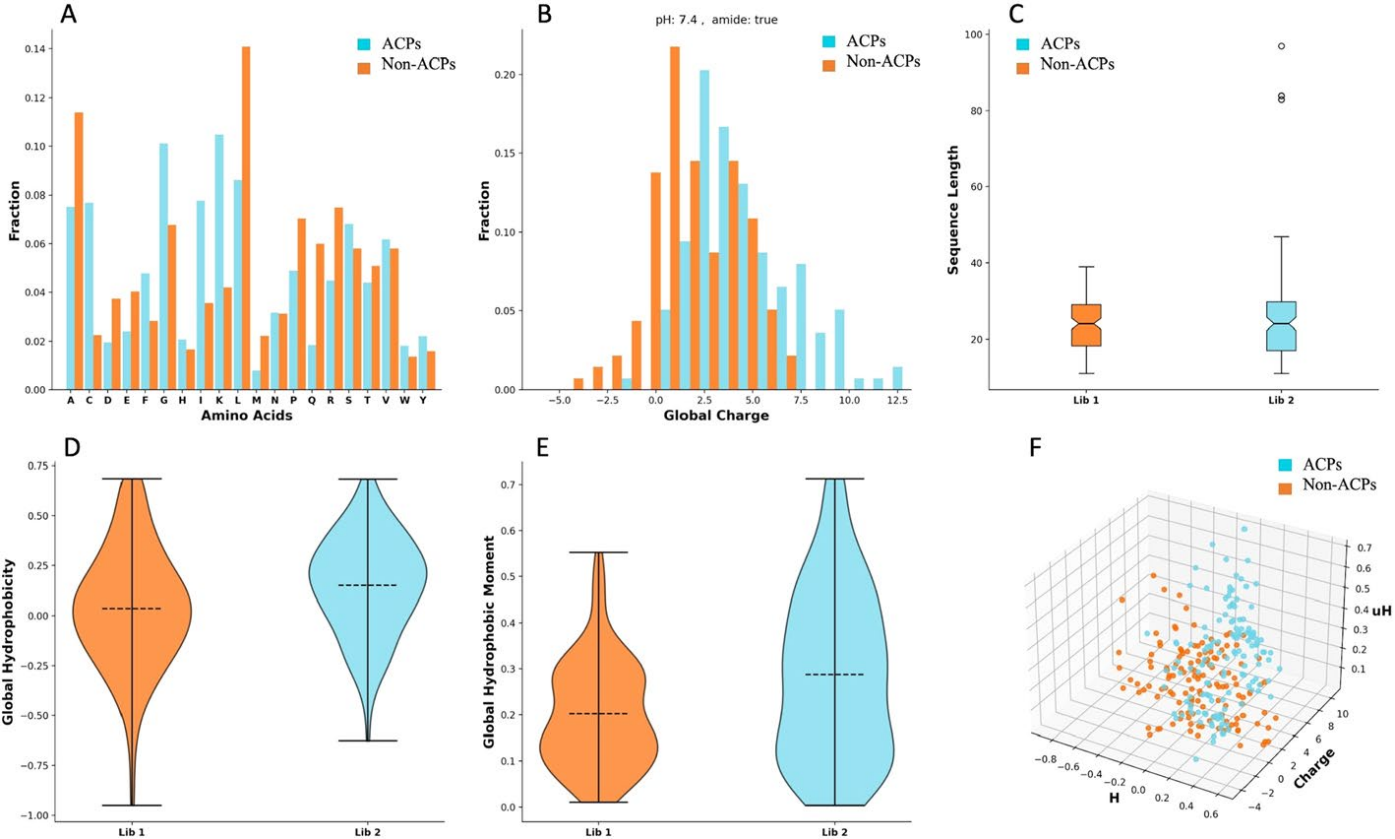
ACPs and non-ACPs datasets were compared and represented with a summary plot. **A** AA frequency distribution plot shows a significant difference in the frequency distribution of G, I, K, and L AA between ACPs and non-ACPs. **B** Global charge distribution shows a higher positive charge among the ACPs, while overall higher negative charge occurs among non-ACPs sequences. **C** There are no significant differences observed in the length distribution of ACPs and non-ACPs, except few outliers. **D** ACPs and non-ACPs show differences in global hydrophobicity. **E** A relatively smaller hydrophobic moment has been observed in the non-ACPs in comparison to the ACPs. **F** 3D scatter plot of global hydrophobicity, global hydrophobic movement and global charge showed separation between ACPs and non-ACPs

**Fig. 5:**
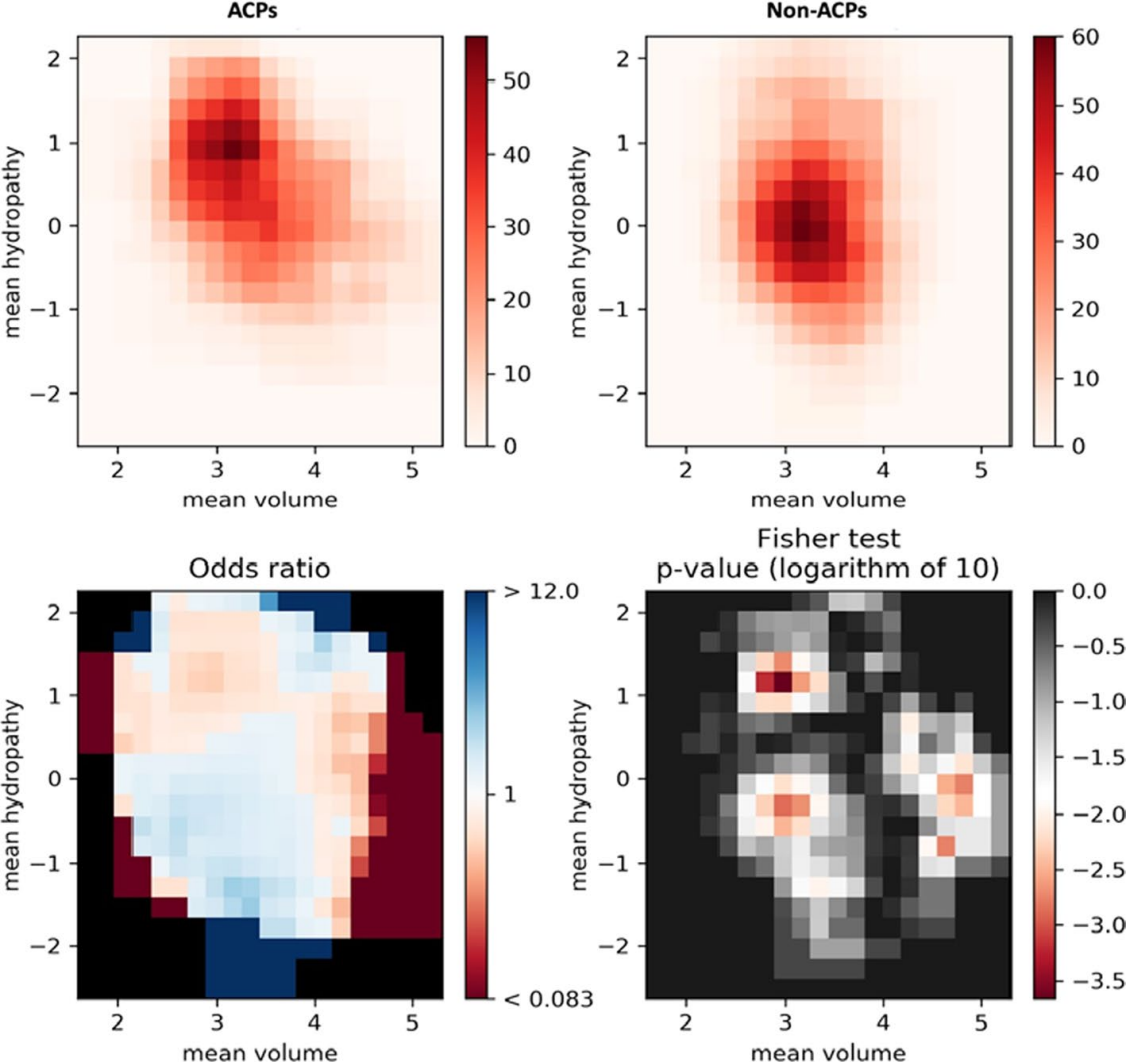
Feature space visualization of ACPs and non-ACPs. ACPs and non-ACPs in the feature space represented by their mean hydropathy and AA volume. The sequences with larger hydrophobic AA are more frequent in ACPs in comparison to non-ACPs

Figure 2 describes the length distribution of the ACP and non-ACP sequences. Mean lengths of ACPs and non-ACPs are observed somewhere in the range of 32–40 AA. The sequence similarity network calculated by Levenshtein distance algorithms shows two compact clusters in the ACP dataset, conversely, a comparatively scattered network is observed in the case of the non-ACP dataset. The sequence similarity network shows relatively fewer diverse sequences in the ACP data set in comparison to the non-ACP sequence (Fig. 3). A detailed summary plot, which compares ACPs and non-ACPs based on their AA fraction, global hydrophobicity, global hydrophobic movement, and global charge, was created with the help of the “PDAUG Peptide Sequence Analysis” tool. The results suggest a significant difference in the frequency distribution of G, I, K, and L AA between the two datasets Fig. 4A. Global charge distribution, Fig. 4B, shows that ACPs depict a relatively higher positive global charge in comparison to non-ACPs, which tend to hold a relatively higher global negative charge. In addition to this, higher global hydrophobicity and hydrophobic movement have been observed in Fig. 4D, E respectively, in the case of ACPs. ACPs and non-ACPs show separation when plotted on the basis of their global hydrophobicity, global hydrophobic movement, and global change on a 3D scatter plot, Fig. 4F. Fisher’s test was used to explore the feature space expressed by hydropathy and the volume of AA. The example data set depicts a significant over-representation of ACP sequences with larger hydrophobic AA. On the other hand, in Fig. 5 we can clearly observe that smaller hydrophilic residues are more frequent among sequences present in non-ACP groups.

### ML modeling results

**Fig. 6:**
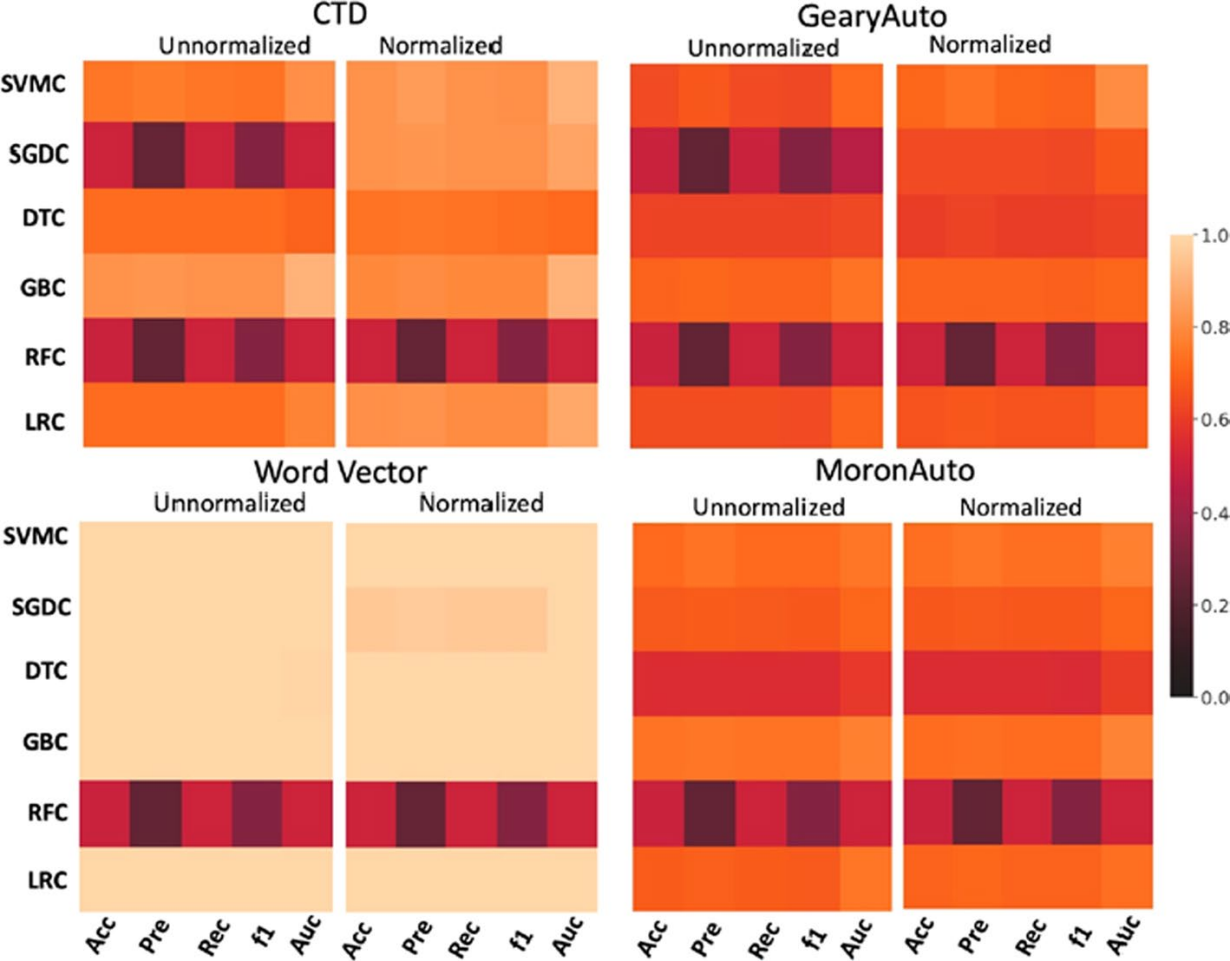
Assessment of the ML algorithms trained on four descriptor sets. Different performance measures for accuracy, precision, recall, F1 score and mean AUC were calculated for six different algorithms with and without z-scaling normalization. Results suggest that the models trained on the word vector descriptors perform superior to the models trained on other descriptors

The accuracy and performance of all algorithms greatly depend upon the robustness of the training parameters, size and complexity of the test dataset. Supervised classification methods have been commonly used to construct statistical models to predict across an unknown dataset based on a trained model. In this study, we have used common performance measures, which includes, precision, recall, f1, measure, accuracy and AUC, to evaluate the performance of the enlisted ML algorithms. Details of these performance measures have been already included in the implementation section. Results suggest that data normalization plays an important role in ML modeling and improves the performance of various ML algorithms. In Fig. 6, we can clearly observe that normalization significantly improves the performance of almost all of the algorithms except RFC, which is not affected by normalization in this study (Fig. 6). The descriptor set is an important factor that plays a crucial role in the performance of the ML algorithms. Here we examined the effect of different descriptors on an ML algorithm and vice versa. First, the impact of descriptors on the ML performance was assessed, and we found that ML algorithms exhibit comparatively higher performance when trained on CTD descriptors, in comparison to Moran and Geary Autocorrelation descriptors. Additionally, a relatively higher positive effect of normalization was also observed if the model was trained on CTD descriptors in comparison to the models trained on other two descriptor sets (Fig. 6). We found that SGDC is very sensitive to normalization and exhibits a significant improvement in the performance when trained on different descriptor sets. Conversely, as described earlier, RFC shows less sensitivity to normalization and remains almost unaffected when trained on different descriptor sets. The other three algorithms, GBC, SVM, and LRC showed improved (Fig. 6) performance after normalization in the case of all the three previously mentioned descriptor sets. Interestingly, Word Vector descriptors, which are commonly used in natural language processing and adopted here to calculate text-based descriptors for ML modeling, outperformed models trained on other descriptor sets in order to classify peptide sequences. We can clearly observe in Fig. 6 that almost all of the algorithms depict model accuracy close to 1.0 for each accuracy measure when trained on the Word Vector descriptor set, in comparison to the other three descriptor sets (Fig. 6). Results clearly indicate that the Word Vector descriptors outperformed all the other three descriptor sets, and exhibits relatively high accuracy in ML model building for this study. Workflows for each analysis, including ML modeling with the sequence-based descriptors (Additional file 1: Fig. S1), ML modeling with word vector descriptors (Additional file 1: Fig. S2), and peptide library analysis workflow (Additional file 1: Fig. S3), are provided along with the example dataset as supplementary material.

Many efforts have been made by researchers to provide various web-based tools and programming libraries such as ifeature server (https://ifeature.erc.monash.edu/) [9], which provides a web interface and Python package to calculate descriptors. Programming libraries such as PyBioMed [16], modLamp [40], PepFun [43], and PyDPI [8] provide various functions to analyze data and solve challenges in peptide research. Public web servers are excellent in terms of accessibility and ease of use,however, they are often not very helpful in creating complex workflows and pipelines. On the other hand, programming libraries provide freedom to create complex pipelines and workflows, but due to the lack of programming expertise, it's not easy for everyone to use them [20]. Since PDAUG is developed on top of the Galaxy platform, it addresses all of the above-mentioned challenges. PDAUG is equipped with standard and advanced algorithms for peptide library generation, descriptor calculation, ML modeling, and data visualization. These essential functionalities have been provided in a single toolset in such a way that they can be utilized to create complex, flexible, and reproducible workflows without the knowledge of programming nor the requirement of any other resources. In addition to this, users can combine other Galaxy community tools from the ToolShed in PDAUG workflows to extend their analysis.

However, despite the above-mentioned features, we believe that there are some limitations of the PDAUG toolset that can be addressed in the future, thus offering scope for new researchers and for us to amend and improve the toolset. Deep learning algorithms are increasingly popular, and in several studies, they outperformed classical ML algorithms. Currently, we have not included any deep learning methods in this toolset. Similarly, 3D structure prediction is currently restricted to peptides with 3 AA due to limitations of the underlying algorithm. In our current implementation, descriptor calculation methods rely only on the sequence-based features, therefore, there is an absence of a method that can directly account for structural features of a peptide sequence.

## Conclusion

PDAUG leverages the Galaxy platform to provide a user-friendly, reproducible peptide analysis environment. Researchers are able to assess the impact of differing tools, methods and algorithms, and can share and distribute their results and workflows. This toolset provides researchers with access to GUI based tools for peptide library generation, feature analysis, data visualization and plotting, ML modeling, and dataset retrieval. PDAUG is released as an open-source toolset under the MIT license with source code available from https://github.com/jaidevjoshi83/pdaug. Installation of PDAUG into a researcher’s Galaxy instance can be achieved using a point-and-click interface from the ToolShed. A Docker image containing a PDAUG Galaxy system can also be obtained from https://hub.docker.com/r/jayadevjoshi12/galaxy_pdaug (https://github.com/jaidevjoshi83/docker_pdaug). Two interactive tutorials featuring this toolset, including workflows and sample datasets, combined with a detailed explanation of various tools, are available from https://training.galaxyproject.org/training-material/topics/proteomics/tutorials/peptide-library-data-analysis/tutorial.html and https://training.galaxyproject.org/training-material/topics/proteomics/tutorials/ml-modeling-of-anti-cancer-peptides/tutorial.html. A PDF version of these tutorials is also provided within the supplementary data.

## Availability and requirements

- Project name: PDAUG
- Project home page: https://github.com/jaidevjoshi83/pdaug.git
- Operating system(s): Platform independent
- Programming language: Python
- Other requirements: Galaxy
- License: MIT

## Supplementary Information

**Additional file 1: Figures.**Figure describing ML modeling workflow to perform ML modeling based on word2vec descriptors. Figure describing ML workflow to perform ML modeling based on CTD, GearyAuto and MoranAuto descriptors. Figure describing workflow to generate summary plot, Fisher’s plot, sequence similarity network, and length distribution plots. Figure of summary plot workflow.

**Additional file 2.** Anti-cancer peptides. FASTA file containing 138 Anti-Cancer Peptides collected from previously published experimental studies.

**Additional file 3.** Non-anti-cancer peptides. FASTA file containing 138 non-Anti-Cancer Peptides collected from previously published experimental studies.

**Additional file 4.** Class labeled ACPs and non-ACPs. Tabular file containing class labeled ACPs and non-ACPs.

**Additional file 5.** Word Vector Model. A pre-calculated word-vector model to calculate word2vec descriptors.

**Additional file 6.** Galaxy tutorial for ML Modeling of ACPs. A detailed tutorial to perform ML modeling of ACPs and non-ACPs using PDAUG.

**Additional file 7.** Galaxy tutorial for peptide library analysis. A detailed tutorial to perform a basic peptide library analysis and data visualization.

**Additional file 8.** Galaxy workflow file for figures. Ready to use Galaxy workflow file to reproduce peptide library analysis and figures, presented in the manuscript.

**Additional file 9.** Galaxy workflow file for ML modeling. Ready to use Galaxy workflow file to reproduce ML modeling based on CTD, GearyAuto and MoranAuto descriptors.

**Additional file 10.** Galaxy workflow file for word2Vec descriptor-based ML modeling. Ready to use Galaxy workflow file to reproduce ML modeling based on word2Vec descriptors.

**Additional file 11: Table S1.** Mathematical equations for descriptors. Table includes important mathematical equations for the implemented descriptors.

## Data Availability

Source code is available from https://github.com/jaidevjoshi83/pdaug. Installation of PDAUG into a researcher’s Galaxy instance can be achieved using the point-and-click interface from the ToolShed. A Docker image containing a PDAUG Galaxy system can also be obtained from https://hub.docker.com/r/jayadevjoshi12/galaxy_pdaug (https://github.com/jaidevjoshi83/docker_pdaug). Two interactive tutorials featuring this toolset, including workflows and sample datasets, combined with a detailed explanation of various tools, are available from https://training.galaxyproject.org/training-material/topics/proteomics/tutorials/peptide-library-data-analysis/tutorial.html and https://training.galaxyproject.org/training-material/topics/proteomics/tutorials/ml-modeling-of-anti-cancer-peptides/tutorial.html. A PDF version of these tutorials is also provided within the supplementary data. Data used in the example analysis is available as supplementary data and from Zenodo (https://doi.org/10.5281/zenodo.4111092).

## Abbreviations

3D: Three dimension
AA: Amino acid
ACP: Anticancer peptide
AMP: Antimicrobial peptides
APD: Antimicrobial peptide database
APD3: Antimicrobial peptide-3
AUC: Area under curve
CAMP: Collection of antimicrobial peptides database
CBOW: Continuous bag of words
CD: Circular dichroism
CTD: Composition, transition, distribution descriptors
DNA: Deoxy-ribonucleic acid
DTC: Decision-tree classifier
FN: False negative
FP: False positive
GBC: Gradient boosting classifier
GNBC: Gaussian Naïve–Bayes classifier
GUI: Graphical user interface
HTTP: Helical transmembrane peptides
IO: Input/output
KNBC: K-nearest neighbor classifier
LRC: Logistic regression classifier
MHC: Major histocompatibility complex
ML: Machine learning
MLP: Multilayer perceptron
PDAUG: Peptide design and analysis under galaxy
PPV: Probability of positive values
RFC: Random forest classifier
RNA: Ribonucleic acid
ROC: Receiver operating characteristic
SGDC: Stochastic gradient descent classifier
SVMC: Support vector machines classifier
TN: True negative
TP: True positive
XML: EXtensible markup language
