# Supplementary material for: PDAUG: a Galaxy based toolset for peptide library analysis, visualization, and machine learning modeling
Source: BMC Bioinformatics. 2022 May 28;23:197. doi: 10.1186/s12859-022-04727-6 (PMC9148462; doi:10.1186/s12859-022-04727-6)
Supplement: Supplementary file 1 — Additional file 1: Figures.Figure describing ML modeling workflow to perform ML modeling based on word2vec descriptors. Figure describing ML workflow to perform ML modeling based on CTD, GearyAuto and MoranAuto descriptors. Figure describing workflow to generate summary plot, Fisher’s plot, sequence similarity network, and length distribution plots. Figure of summary plot workflow. [file 12859_2022_4727_MOESM1_ESM.pdf]

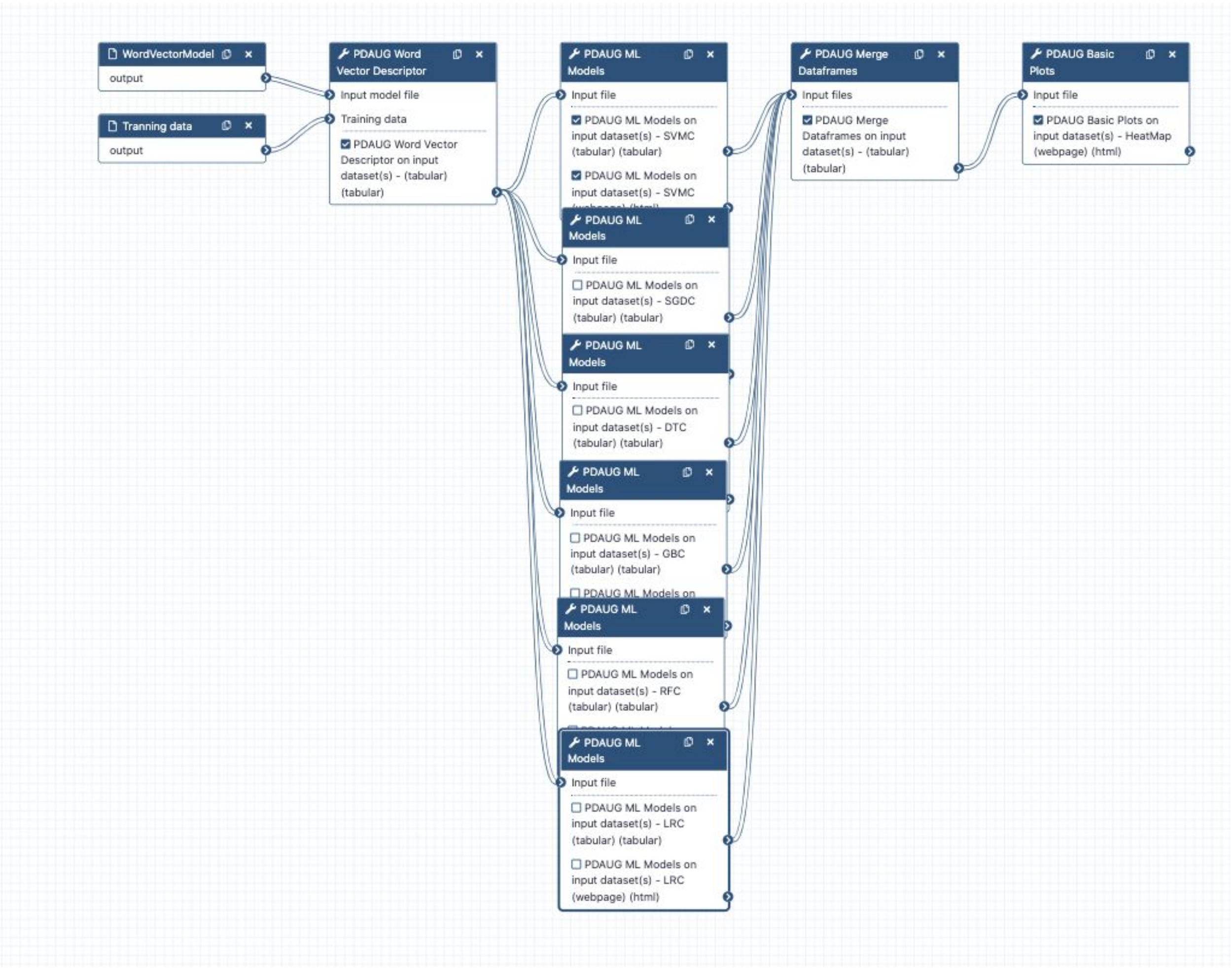

Supplementary Figure 1. Workflow to perform machine learning modeling based on word vector descriptors.

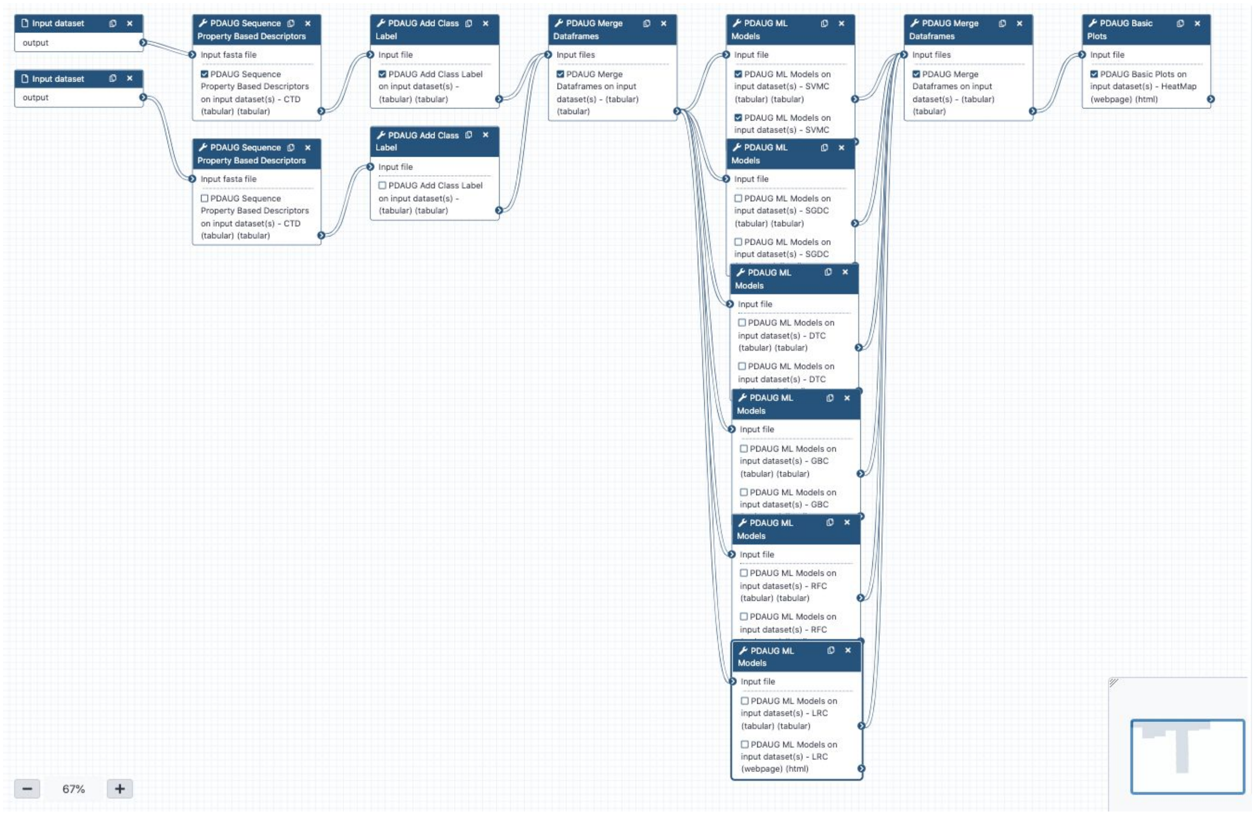

Supplementary Figure 2. Workflow to perform machine learning modeling based on CTD descriptors.

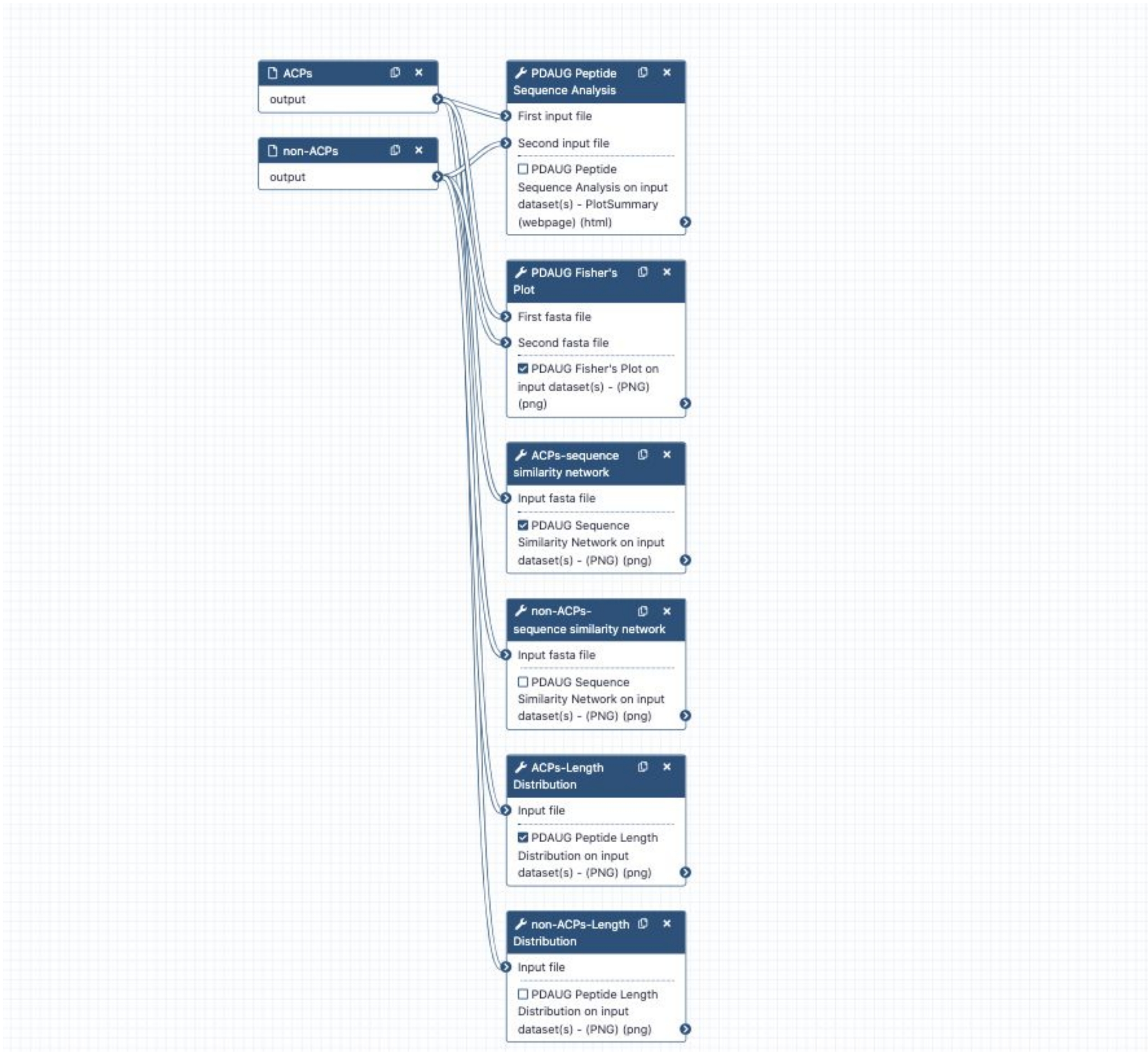

Supplementary Figure 3. Workflow to generate summary plot, Fisher's plot, sequence similarity network, and length distribution plot.
