# Supplementary material for: PDAUG: a Galaxy based toolset for peptide library analysis, visualization, and machine learning modeling
Source: BMC Bioinformatics. 2022 May 28;23:197. doi: 10.1186/s12859-022-04727-6 (PMC9148462; doi:10.1186/s12859-022-04727-6)
Supplement: Supplementary file 11 — Additional file 11: Table S1. Mathematical equations for descriptors. Table includes important mathematical equations for the implemented descriptors. [file 12859_2022_4727_MOESM11_ESM.pdf]

Supplementary Table 1. Important mathematical equations have been included for the implemented descriptors.

Descriptor name	Formula
Charge Density	$CD = C/D$, C = Charge, D = Density
Isoelectric point	pI = ½(pK_{a1} + pKa2)
Instabiliity Index	$II = \frac{10}{L} \times \sum DIWV(x[i] \times [i+1])$<br>DIWV-Di-peptide weight value<br>L length of sequence.<br>I Position
GetAAComp	$f(t) = \frac{N(t)}{N}, \quad t \in \{A, C, D, ..Y\}$<br>N is number of Amino Acids, t is type $A, C, D, ..Y$
GetDPComp	$D(r,s) = \frac{Nrs}{N-1}, \quad r,s \in \{A, C, D, ..Y\}$<br>*Nrs* is dipeptides represented by amino acid types *r* and *s*.
GetTPComp	$D(r,s,t) = \frac{Nrst}{N-2}, \quad r,s,t \in \{A, C, D, ..Y\}$<br>*Nrst* tripeptides represented by amino acid types *r, s and t.*
GetMoranAuto	$AC(d) = \sum_{i=1}^{N-d} P_i \times P_{i+d}, \quad d = 1, 2, ..., nlag$<br>*d* is the lag of the autocorrelation,<br>*nlag* is the maximum value of the lag<br>*Pi* and *Pi+d* are the properties of the amino acids at positions *i* and *i* + *d*, respectively.
GetMoreauBrotoAuto	$ATS(d) = \frac{AC(d)}{N-d}, \quad d = 1, 2, ..., nlag$<br>*D N and nlag* have the same definitions as described above
GetGearAuto	$C(d) = \frac{\frac{1}{2(N-d)} \sum_{i=1}^{N-d} (P_i - P_{i+d})^2}{\frac{1}{N-1} \sum_{i=1}^{N} (P_i - \bar{P}')^2}, \quad d = 1, 2, ..., nlag$

	d, *P*, *Pi*, *Pi+d* and *nlag* same as above.
GetCTD	$$C(r) = \frac{N(r)}{N}, \quad r \in \{polar, neutral, hydrophobic\}$$ *N(r)* is the number of amino acid. *r* is the type and *N* is the length of the sequence.
GetPAAC	$$H_1(i) = \frac{H_1^o(i) - \frac{1}{20}\sum_{i=1}^{20} H_1^o(i)}{\sqrt{\frac{\sum_{i=1}^{20}[H_1^o(i) - \frac{1}{20}\sum_{i=1}^{20} H_1^o(i)]^2}{20}}}$$ $H_1^0(1)$, $H_2^0(1)$ and $M^o(i)$ are original hydrophobicity values for i = 1,2,3,4…20 amino Acids.
GetSOCN	$$\tau_d = \sum_{i=1}^{N-d} (d_{i,i+d})^2, \quad d = 1, 2, 3, ..., nlag$$ *di, i+d* distance between the two amino acids at position *i* and *i + d.* *nlag* denotes the maximum value of the lag, *N* is the length of a protein or peptide sequence.
GetQSO	$$X_r = \frac{f_r}{\sum_{r=1}^{20} f_r + w\sum_{d=1}^{nlag} \tau_d}, \quad r = 1, 2, ..., 20$$ *fr* is the normalized occurrence of amino acid, *r is the type, w is* weighting factor (*w* = 0.1), *nlag* and τ_d same as described above.
GetTriad	$$d_i = \frac{f_i - \min\{f_1, f_2, ..., f_{343}\}}{\max\{f_1, f_2, ..., f_{343}\}}$$ *fi*, the value of the *i*-th dimension of number vectors correspond to the vector space of a sequence features and each feature.
Calculate AutoCor	Standardization of amino acid

	$$P_r = \frac{P_r - \bar{P}}{\sigma}$$ Auto correlation $$\bar{P} = \frac{\sum_{r=1}^{20} P_r}{20} \quad \text{and} \quad \sigma = \sqrt{\frac{1}{2}\sum_{r=1}^{20}(P_r - \bar{P})^2}$$ P is the property and P¯ is the average of the property of the 20 amino acids
